# S179D Prolactin Sensitizes Human PC3 Prostate Cancer Xenografts to Anti-tumor Effects of Well-Tolerated Doses of Calcitriol

**DOI:** 10.26502/jcsct.5079085

**Published:** 2020-10-05

**Authors:** Christopher T. Holland, Joffrey Hsu, Ameae M. Walker

**Affiliations:** Division of Biomedical Sciences, School of Medicine, University of California, Riverside, CA 92521, USA

**Keywords:** Selective Prolactin Receptor Modulator, synergy, Apoptosis, Anti-angiogenesis, Dual therapy

## Abstract

Calcitriol has been shown to have multiple anti-prostate cancer effects both *in vitro* and in xenograft models, and associations between low levels of calcitriol and more aggressive forms of prostate cancer have been observed clinically. However, the concentrations of calcitriol required to have a substantive anti-cancer effect *in vivo* are toxic. In previous work, we had observed that the selective prolactin receptor modulator, S179D PRL, sensitized prostate cancer cells *in vitro* to physiological concentrations of calcitriol through an ability to increase expression of the vitamin D receptor. Here, we have investigated whether administration of S179D PRL would likewise sensitize androgen-insensitive human PC3 xenografts *in vivo* and do so without inducing tissue damage akin to hypervitaminosis D. Using low concentrations of both S179D PRL (250 ng/h) and calcitriol (up to 220 pg/h), we found no effect of each alone or in combination on the growth rate of tumors. However, there was increased central tumor death with their combination that was more than additive at 250 ng S179D PRL and 220 pg calcitriol per hour. Both S179D PRL and calcitriol alone were antiangiogenic, but their antiangiogenic effects were not additive. Also, both S179D PRL and calcitriol alone increased the number of apoptotic cells in tumor sections, but their combination reduced the number, suggesting more effective clearance of apoptotic cells. Histopathology of the livers and kidneys showed no changes consistent with hypervitaminosis D. We conclude that dual therapy holds promise as a means to harness the anti-tumor effects of well-tolerated doses of calcitriol.

## 1. Introduction

Prostate cancer, which is the second leading cause of death from cancer among men in the United States, will affect about one man in nine during their lifetime, with a projected 192,000 new cases likely to be diagnosed in the United States throughout 2020 [1]. Initially, most prostate cancers are responsive to androgen deprivation therapy, but eventually, some will progress to a castration resistant state [2].These castration resistant prostate cancers (CRPC) require alternate approaches to treatment, which currently include traditional chemotherapeutics, newer androgen receptor antagonists, and a variety of immunotherapies [3]. While significant progress has been made and there is some survival benefit with the new treatments, there is a lot still to be learned about CRPC. The more we understand, the greater the number of potential treatment avenues that may eventually contribute to substantial survival gains.

There are a variety of hormones and cytokines considered important growth factors for prostate cancer, one of which is the hormone, prolactin (PRL) [4]. Human prostate cells in the healthy and cancerous states express both the hormone, PRL, and three splice variants of the prolactin receptor, a long form (PRLRLF) and two short forms, PRLRSF1a and PRLRSF1b [4]. Thus, in addition to responding to circulating PRL from the pituitary, cells of the prostate can respond to their own PRL, creating an autocrine PRL growth loop [4,5]. Furthermore, PRL is a survival factor for the androgen-deprived prostate [6]. PRL (endocrine or autocrine), acting via the PRLRLF *in vivo*, activates the Janus Kinase 2 - Signal Transducer and Activator of Transcription 5 (Jak 2-Stat5a/b) pathway, preventing apoptosis and allowing the survival of prostate cancer cells [7]. Stat5 activation is also associated with increased metastatic spread [7, 8], and elevated Stat5 levels are correlated with a decrease in E-cadherin expression and increased migratory capacity. Inhibition of Stat5 activation by contrast, increases E-cadherin and p21, and leads to activation of the extrinsic apoptotic pathway, ultimately leading to cancer cell death [8, 9].

S179D PRL is a molecular mimic of naturally phosphorylated human prolactin [10]. Although it binds to and activates the PRLRLF, the resulting signal cascade differs from that produced by unmodified PRL. Most importantly, there is inhibition of the activation of Stat5 and prolonged and sustained activation of ERK 1/2 leading to increased expression of the vitamin D receptor (VDR), E-cadherin, the PRLR SF1b and the cell cycle-inhibiting protein, p21 [11–14]. Furthermore, by interrupting the PRL autocrine growth loop, S179D PRL delays the appearance and decreases the growth of human prostate cancer xenografts [5]. The VDR is expressed in the DU145 and PC-3 prostate cancer cell lines and treatment with calcitriol inhibits proliferation and maintains differentiation of these cells [15,16]. The VDR is an important positive regulator of p21 expression and hence an important inhibitor of growth and promoter of apoptosis in prostate cancer cells [12, 17]. When used as an isolated treatment, the doses of calcitriol required to achieve therapeutic effects in prostate cancer increase intestinal absorption of calcium and activate osteoclasts, thereby increasing serum calcium to supraphysiological, toxic levels [18, 19]. Calcium toxicity can cause kidney stones, acute renal failure, altered mental status and cardiac arrhythmias [20]. For these reasons, single agent treatment with calcitriol is not suitable for clinical applications. Using prostate cancer cell lines *in vitro,* we have previously shown that S179D PRL sensitizes DU145 and PC-3 cells to the anti-tumor effects of physiological concentrations of calcitriol through its ability to increase expression of the VDR [13]. *In vitro* there was a synergistic reduction in viable cell number when cells were treated with S179D PRL in conjunction with physiological doses of calcitriol [13].

The purpose of the current study was to determine whether co-treatment with S179D PRL and non-toxic levels of calcitriol would produce a similar therapeutic synergy *in vivo*. In addition to direct effects on tumor cells, there was the possibility of a further synergy since both S179D PRL and calcitriol have been shown to be antiangiogenic [21,22]. PC-3 cells are a highly metastatic human cell line that, like advanced prostate cancer, is resistant to androgen deprivation therapy [23,24]. We have therefore employed this cell line to test the *in vivo* efficacy of S179D PRL and calcitriol co-treatment. Using tumor cell death as the measure, we report a more than additive effect from co-treatment with well-tolerated doses of calcitriol together with S179D PRL.

## 2. Materials and Methods

### 2.1 Human prostate cancer xenografts and treatments

All procedures involving animals were approved by the University of California, Riverside Institutional Animal Care and Use Committee. PC3 human prostate cancer cells were freshly obtained from American Type Culture Collection to ensure their authenticity. They were cultured in Modified Eagle’s medium supplemented with 10% fetal bovine serum. Eight week-old, male, athymic nu/nu mice were inoculated with 5 × 10^6^ PC-3 cells in 50% Matrigel (Becton, Dickinson and Company, Franklin Lakes, NJ) subcutaneously on Day 0 of the study. When overall mean relative tumor size reached 100mg ± 10%, mice were distributed into the treatment groups such that tumor size means were equal. Because 100mg was not an actual, but a calculated tumor weight based on the formula 1/2 (Length × Width2) and an assumption of a density of 1, we have labeled tumor growth graphs in terms of relative units, with the calculated 100mg equal to 100 Units (U). Each group had nine mice at the outset of treatment. Alzet™ (Alza, Palo Alto, CA) osmotic pumps were implanted subcutaneously between the scapulae on day 12, which is when the tumors reached 100U ± 10%. Day 12 was considered to be the first day of treatment, even though it takes approximately 3 days at a rate of 250ng/h, for levels of S179D PRL in the circulation to stabilize [5]. S179D PRL was produced as previously described and was dissolved in Dulbecco’s phosphate buffered saline [10]. Calcitriol was a gift from Anthony W. Norman (Division of Biomedical Sciences, University of California, Riverside) and was diluted in propylene glycol so as to have appropriate density for delivery by minipump. For hormone combinations, both hormones were present in the same pump. Control animals received a baseline amount of calcitriol (4.25 pg/h) in propylene glycol to ensure equivalent handling. By keeping the dose of S179D PRL low and constant, we could test for additive or more than additive effects with increasing doses of calcitriol.

Clinical health of the animals was recorded daily and body weights and tumor measurements were recorded twice weekly. Measurements were conducted in a blinded fashion, with personnel recording results according to treatment group number. Mice found to be moribund, likely as a result of inadvertent intravascular delivery of some tumor cells at the time of inoculation, were euthanized before the end of the study and were not included in the analyses (~ 1 per group). The pumps are designed to deliver their contents for 28 days, completing the treatment period at day 40. In order to determine any continued post-treatment benefit from therapy, the mice were followed for tumor responses until day 47 when they were euthanized, and tissue specimens collected.

### 2.2 Histological analyses

The tumor, liver and kidney from each animal were fixed in 10% buffered formalin, followed by processing for histological sectioning. Five micron serial sections were cut, stained with hematoxylin and eosin by standard protocol and mounted for light microscopic examination. For each of the quantitative analyses below, at least three central sections from each tumor were examined in detail. Images of the stained sections were captured using a Nikon Eclipse E600 microscope equipped with a Paxcam3 digital camera and Pax-it imaging software. Image analysis was conducted using the open source NIH imaging program, *Image-J*. All image analyses were conducted in a blinded fashion by coding of the treatment groups.

### 2.3 Tumor cell death

Multiple digital images of stained sections through the point of greatest diameter of each tumor were captured at a magnification of 100x and compiled to reconstruct the entire tumor section using Adobe Photoshop. The total tumor area and area of evident acellular material were measured.

### 2.4 Angiogenesis surrounding the tumor

Using tumor sections at the point of greatest tumor circumference, photographs were taken at a magnification of 200x. All vessels within one field of view (FOV) from the tumor margin were captured: the slide was oriented to ensure the FOV was composed of twenty percent tumor margin and stromal tissue surrounding the tumor, with the other eighty percent incorporating the tumor parenchyma. The entire circumference of the tumor margin was analyzed by stitching the images together. Microvessels of all kinds (arterioles, venules, capillaries) were counted using a slightly modified version of the intratumor-microvessel density method of Weidner *et al.* [25]; morphology, rather than the presence of luminal red blood cells, was used to identify vessels.

### 2.5 Apoptotic index

The greatest number of apoptotic cells was evident at the junction between the tumor parenchyma and the central tumor acellular material. For this analysis, fifty percent of the FOV incorporated the acellular area and fifty percent the tumor parenchyma. Images of this margin were stitched together to ensure the entire circumference was analyzed. Apoptotic cells were counted using a systematic sampling protocol with guidelines outlined by Van der Shepop *et al.* [26]. Apoptotic cells were identified by condensed chromatin, nuclear remnants and retracted cytoplasm. Cells undergoing both early and late apoptosis were included in the count.

### 2.6 Analysis of potential organ damage

Liver specimens were serially sectioned and examined for any evidence of hepatocyte disruption, loss of nuclei, liver fibrosis, cirrhosis, or inflammatory changes. Kidney tissue was analyzed for any evidence of tubular, glomerular or interstitial changes indicative of kidney damage.

### 2.7 Statistical analysis and graphs

Mann-Whitney or ANOVA with post-tests were used to determine whether differences existed among all or specific treatment groups. Results were considered significant with a *p-*value of less than 0.05. The minimal number of animals per group was seven, but group size was usually 8. Graphs and statistical analyses were generated using Graphpad Prism software.

## 3. Results

### 3.1 Effect of S179D PRL and calcitriol on relative tumor size

**Figure 1:**
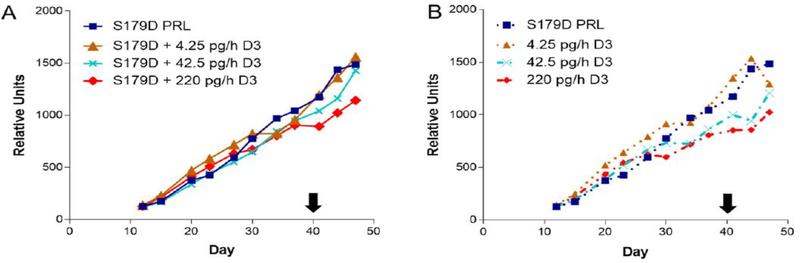
Tumor size as a function of time and treatment. Treatments began on day 12 after mice were assigned to treatment groups such that the mean size of tumors in each group was the same. Treatment concluded at day 40 (black arrow). Each point is the mean of at least 7 animals and usually 8 (see Table 1). (A) S179D PRL alone compared to S179D PRL plus increasing concentrations of D3. (B) S179D PRL alone compared to increasing concentrations of D3 alone.

Previous work [13] showed a synergy between S179D PRL and calcitriol in the promotion of prostate cancer cell death *in vitro*. We therefore anticipated a similar synergy *in vivo*. Tumor size was estimated throughout the study by twice weekly external caliper measurements. For this first graph, the data are divided into those with (Figure 1A) *versus* those without (Figure 1B) S179D PRL. None of the treatments had an overall significant effect on tumor growth, as measured by estimates of tumor volume derived from the external caliper measurements.

### 3.2 Acellular area within tumors

**Figure 2:**
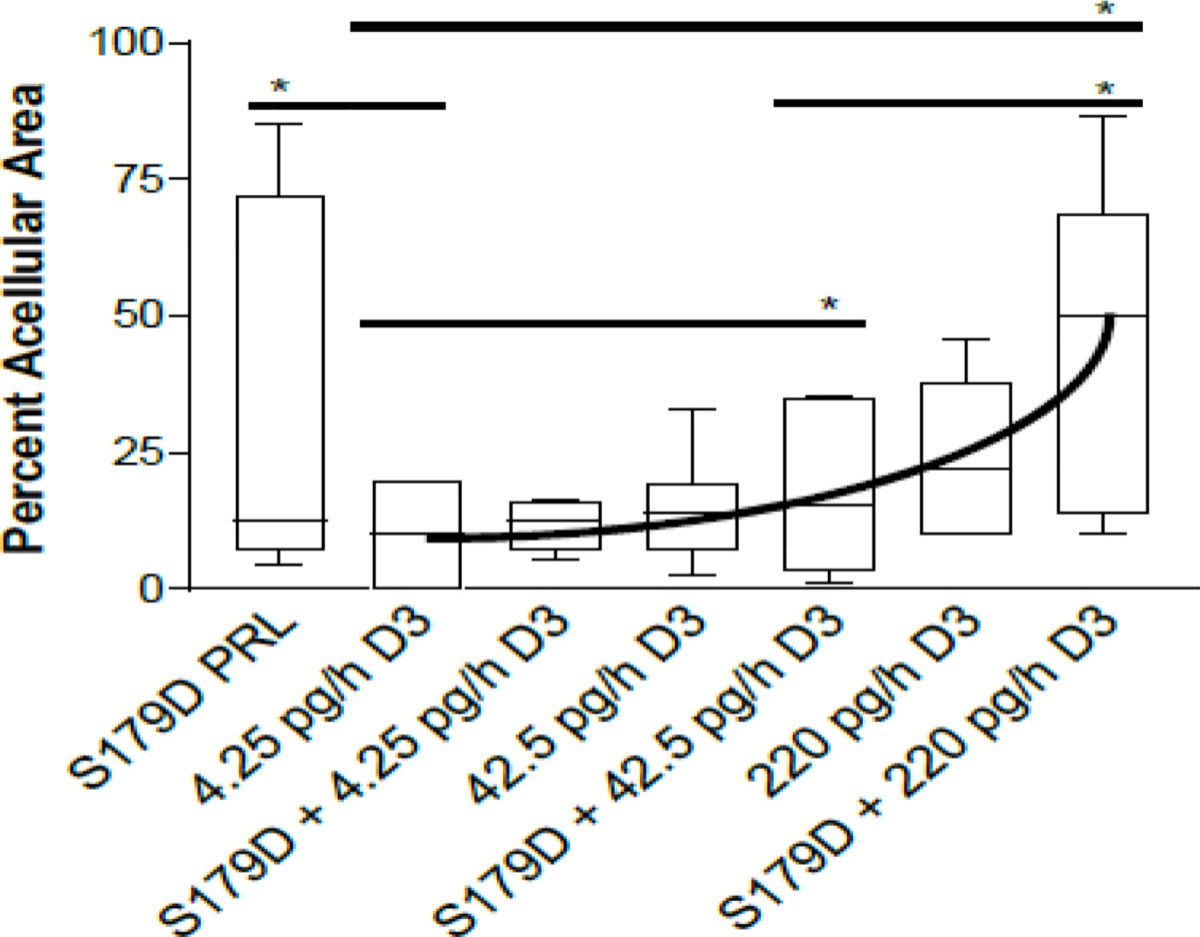
Percent acellular area in the tumors at 47 days. Seven days after the end of the treatments, tumors were sectioned through their largest diameter, micrographs covering the entire area were stitched together and the percent area that was acellular was determined. Data are displayed as box plots with the median and statistically significant differences (*) determined by Mann Whitney indicated. P<0.05.

The percentage of the tumor occupied by acellular material was very variable within groups (Figure 2). However, with focus on the median values, one can appreciate that increasing doses of calcitriol in the presence of S179D PRL showed increasing acellular areas. S179D PRL plus the 220 pg/h dose of calcitriol showed a statistically significant increase in acellular area when compared to S179D PRL plus 42.5 pg/h or control (4.25 pg/h) dose of calcitriol.

At the doses of calcitriol tested by themselves, there were no statistically significant effects observed. Importantly, when comparing the median values for S179D PRL alone and highest dose calcitriol alone, their combination was more than additive, amounting to 50% acellular area in the tumors and suggesting a potential synergistic interplay between the two-hormone treatments, as observed *in vitro* [13].

### 3.3 Angiogenesis in the tumor adnexal regions

**Figure 3:**
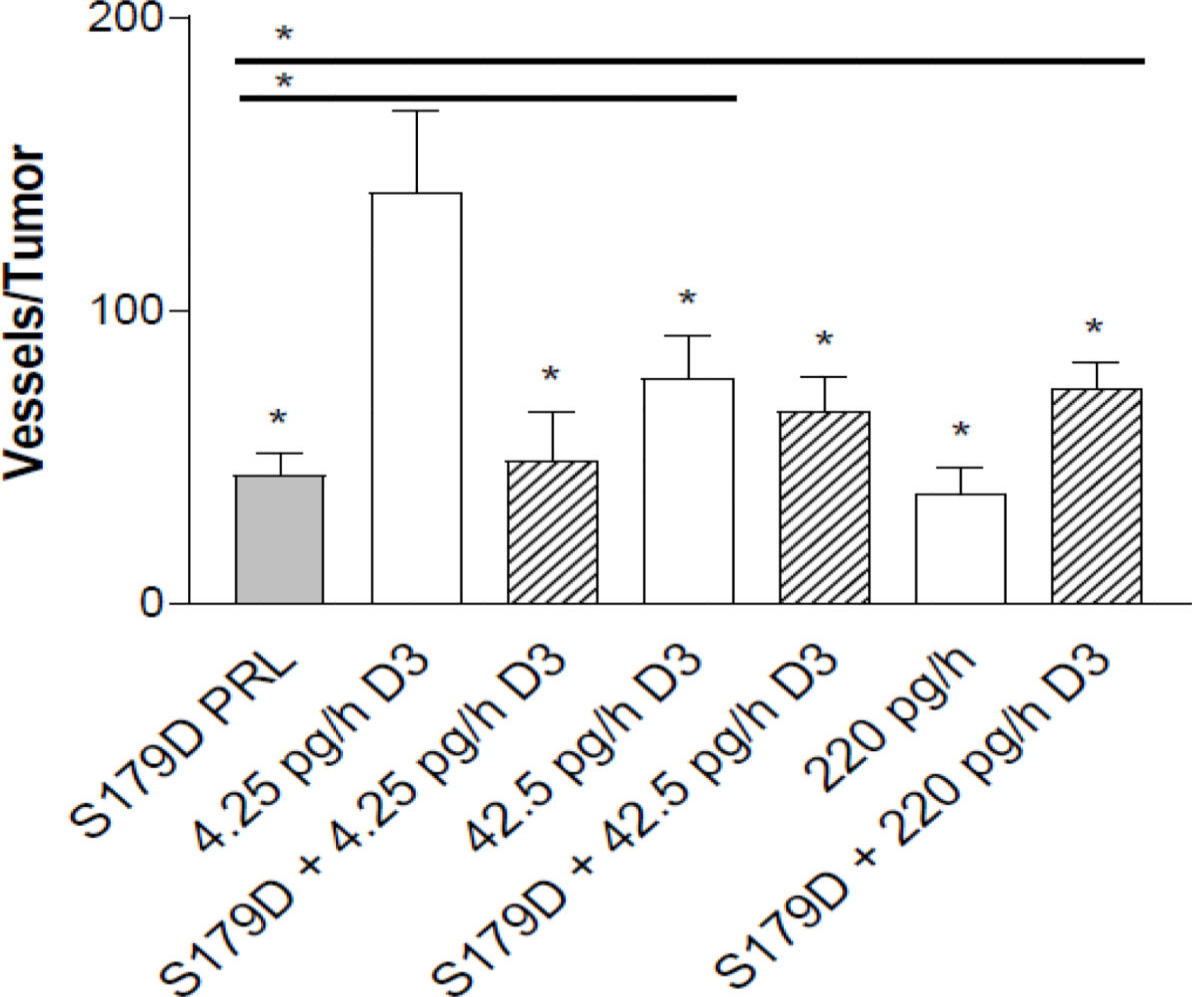
Microvessel density around the tumor at 47 days. Seven days after the end of the treatments, tumors were sectioned through their largest diameter and positioned consistently to count all vessels at the margin of the tumor (see materials and methods). The entire circumference of the tumor margin was analyzed by stitching the images together. *, statistically different from the control or, when with a horizontal line, between the two treatments indicated. P<0.05.

Both calcitriol and S179D PRL have been shown to have antiangiogenic effects [21,22]. By examining microvessel density in the tumor adnexal regions, antiangiogenic effects of each of the hormones were confirmed (Figure 3).

In addition, there was a dose related decrease in microvessel density with increasing concentrations of calcitriol alone. S179D PRL alone and the highest dose calcitriol alone had equivalent antiangiogenic effects, but no additional effect was seen with their combination.

### 3.4 Induction of tumor cell apoptosis

Since both calcitriol and S179D PRL induce apoptosis of prostate cancer cells [5,15,16], the relative number of apoptotic cells at the boundary between the acellular and viable regions of the tumor was assessed.

**Figure 4:**
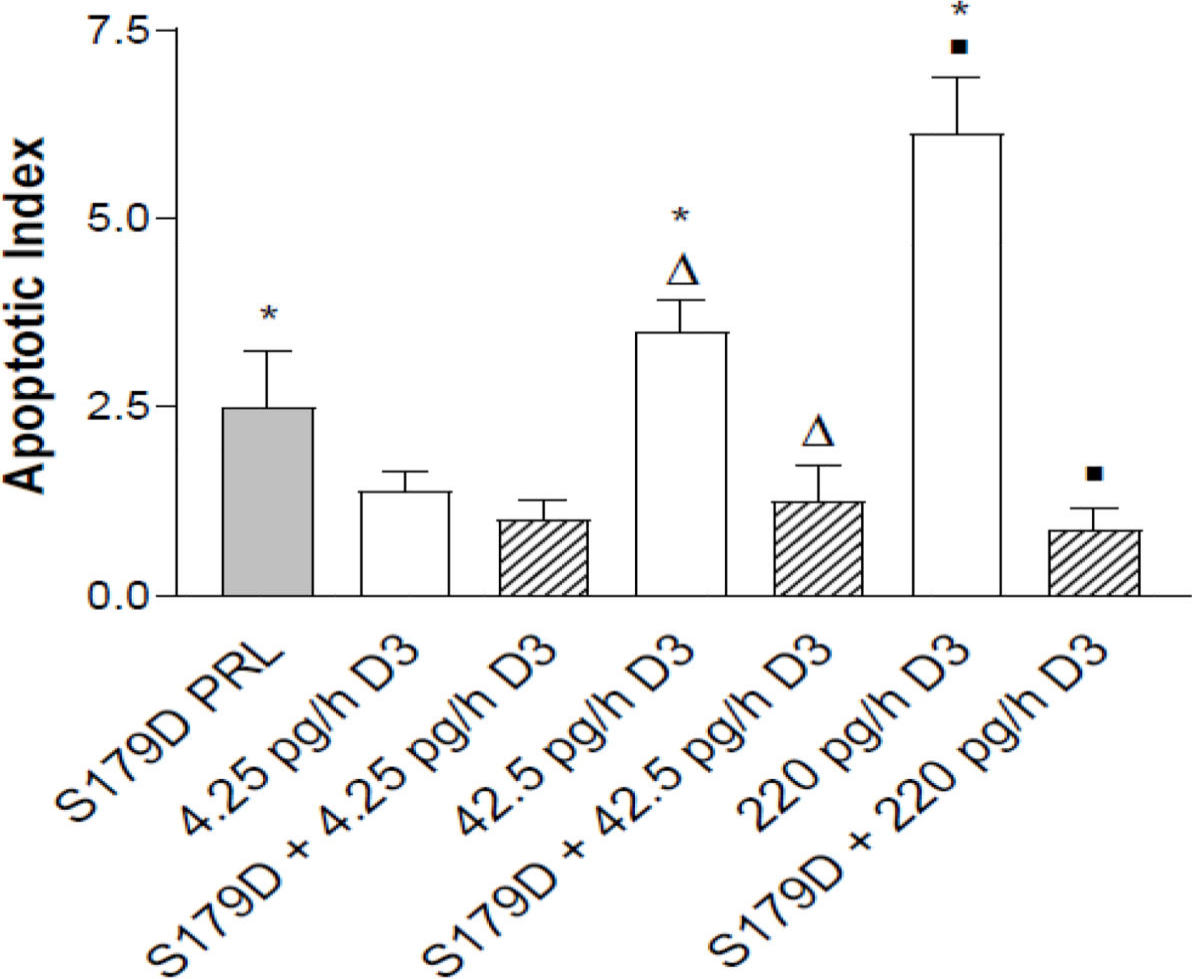
Apoptotic index at the boundary of the acellular region at 47 days. Apoptotic cells were quantified in entire sections through the tumors as described in Materials and Methods. *, different from control; triangles different from each other; squares different from each other. P<0.05.

Calcitriol alone showed a dose-dependent increase in the apoptotic index. In addition, an *in vivo* increase in apoptotic cells in response to S179D PRL was demonstrated (Figure 4). However, contrary to expectations, S179D PRL together with either the 42.5 or 220 pg/day calcitriol decreased the apoptotic index significantly.

### 3.5 Organotoxicity analyses

Because S179D PRL upregulates the VDR [12], it was possible that toxic effects of calcitriol may have occurred at the normally non-toxic doses being examined. Histologically, hypervitaminosis D is characterized by kidney and liver and other damage in organs subject to calcification [20,27]. Our focus was on the liver and kidney both for this reason and because these organs have fairly uniform and high expression of PRLRs [28] and therefore had the greatest potential for upregulation of the VDR in response to S179D PRL.

**Figure 5:**
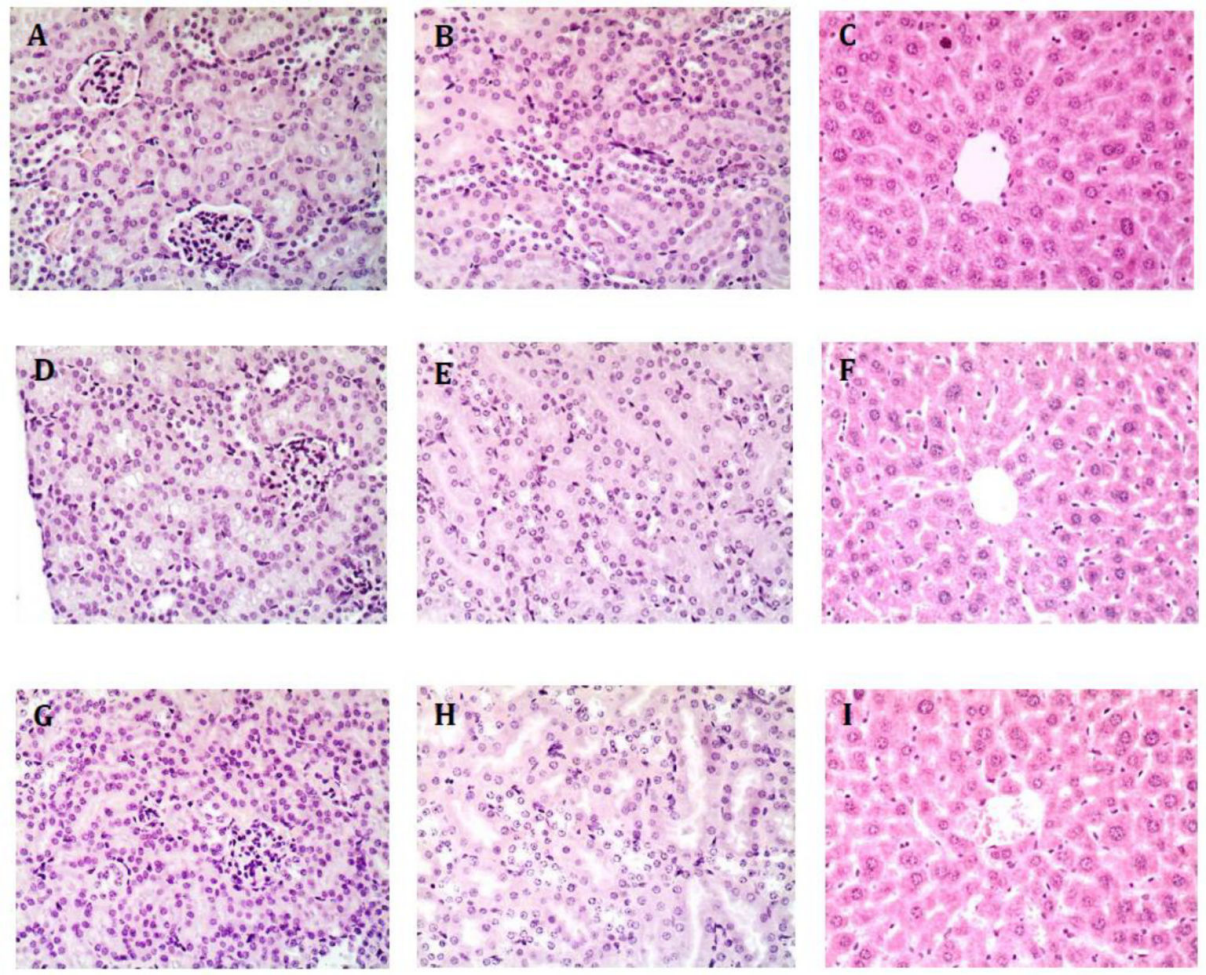
Representative Images of Kidney and Liver from Treated Mice. A-C, control mice receiving 4.25 pg/h calcitriol; D-F, mice receiving 250 ng/h S179D PRL; G-I, mice receiving 220 pg/h calcitriol plus 250 ng/h S179D PRL. Kidneys and livers from each animal in the study were serially sectioned at 47 days and examined for any evidence of damage akin to hypervitaminosis D. No damage was observed. A,D and G show regions of the kidney containing glomeruli; BE and H are higher magnifications focused on kidney tubules; C,F, and I show regions of the liver around a central vein.

Analysis of serial sections of all kidneys from each treatment group showed no glomerular damage or mesangial cell proliferation. All sections showed intact renal tubule cells. Furthermore, there was no evidence of inflammatory changes indicative of interstitial nephritis. Likewise, there was no evidence of hypervitaminosis D-like changes in liver tissue. There was neither microvesicular nor macrovesicular fatty change. The architecture was normal, with intact biliary structures, normal central vein morphology, and no evidence of inflammatory infiltration or fibrosis. In other words, there was no histological evidence of changes characteristic of hypervitaminosis D (Figure 5).

### 3.6 Body weight changes

**Figure 6:**
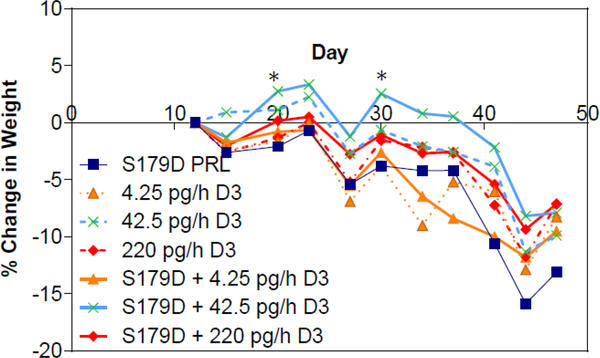
Percent change in body weight as a function of time and treatment. Mice were weighed at the time points indicated. Each point is the mean of at least 7 animals. *, different from other groups at the time points indicated. P<0.05.

Weight loss and cachexia are commonly observed symptoms in patients with advanced metastatic cancers [30]. Only one group of animals maintained their weight during the treatment period (days 12–40) and that was S179D PRL plus the 42.4 pg/h dose of calcitriol, but the difference from other groups was minimal and only statistically significant at two time points (Figure 6). This maintenance of body weight was rapidly lost upon cessation of treatment.

## 4. Discussion

Tumor size is a component of clinical staging in prostate cancer and often stands as a surrogate prognostic factor in outcome [30]. Furthermore, the efficacy of therapeutics is often judged by shrinkage of tumors [31]. However, as the data reported herein suggest, a reduction in tumor size may not always be the best measure of therapeutic efficacy. None of the treatment regimens resulted in a significant reduction in tumor size. Nevertheless, the combination of S179D PRL and the highest dose of calcitriol for only 28 days caused 50% of the tumor volume to become entirely acellular. The appearance was that of classical central necrosis, but cells adjacent to the peripheral viable cells were undergoing apoptosis. Thus, the acellular region may have resulted from a combination of apoptosis and necrosis. Neither the S179D PRL alone nor the calcitriol alone was as effective as their combination, which was more than additive.

Central necrosis occurs as a result of insufficient blood supply to the growing tumor. Blood supply can be assessed by regional and intra-tumor microvessel density [e.g. 32]. Both S179D PRL and calcitriol are anti-angiogenic, in part through antiproliferative and proapoptotic functions in endothelial cells [21, 22, 33]. S179D PRL also decreases production of proangiogenic factors, including vascular endothelial growth factor, basic fibroblast growth factor and hemoxigenase 1 by endothelial cells [21]. Similar activities are attributed to calcitriol and are most dramatically demonstrated in VDR knockout mice. Knockout of the VDR causes endothelial cells to produce increased levels of pro-angiogenic factors, including hypoxia induced growth factor 1β, vascular endothelial growth factor, and angiopoietin-1 [22]. Data in the current study confirm the individual antiangiogenic effects of S179D PRL and calcitriol but show no additive or synergistic effect of their combination, perhaps because of the similarity of their effects on endothelial cells. Since there was no additive antiangiogenic effect, there must be an additional mechanism of action that contributes to central tumor death with dual treatment.

Both S179D PRL and calcitriol have also been shown to promote apoptosis [5, 12, 15, 16, 21, 33]. The apoptotic index was higher than the control for S179D PRL alone and both the 42.5 and 220 pg/h doses of calcitriol alone, but oddly the apoptotic index went down with dual treatment. Rather than indicating opposing effects of each hormone under these circumstances, we propose that this apparent reduction in apoptosis with dual treatment may be due to more rapid clearance of dead cells during the 7-day post-treatment period before the mice were sacrificed. Macrophage disposal of apoptotic cells is a very complex and highly-regulated process. More efficient clearance of apoptotic cells could be the result of effects on the dying cells and release of more macrophage chemoattractants, presentation of more “eat me” signals or, alternatively, could be the result of effects of dual treatment on macrophages to increase the efficiency of engulfment [34]. In support of the latter possibility, macrophages express both the PRLR and VDR. Activation of the PRLR upregulates expression of milkfat globule epidermal growth factor 8, which is crucial for effective phagocytosis of apoptotic cells [35]. This upregulation is mediated through the C/EBPbeta-dependent pathway [35], which can be initiated by sustained activation of ERK1/2 [36]. Moreover, sustained activation of ERK1/2 is a characteristic of signaling from S179D PRL [12, 33]. Thus, S179D PRL may induce milkfat globule epidermal growth factor 8. Furthermore, upregulation of the VDR, which would likely happen in response to S179D PRL, is considered a marker of M2 polarization of macrophages, which polarization is appropriate for the clearance of apoptotic cells [37]. This is clearly an important area for future investigation.

Given that S179D PRL upregulates the VDR and sensitizes prostate cancer cells to calcitriol, it was possible that combination therapy with the relatively low doses of calcitriol used would upregulate the VDR in other tissues and thereby reproduce the toxic effects of hypervitaminosis D. Histological analyses of both liver and kidneys, however, showed no signs of toxicity. Thus, it appears that tumor cells were sensitized without histological evidence of adverse effects in other sensitive tissues. A potential limitation in this analysis was the duration of treatment, but others have observed histological evidence of toxic effects in the liver and kidneys with shorter treatments [27, 38]. We therefore conclude that the treatment regimen was well-tolerated for the duration studied.

A large number of preclinical studies from a variety of laboratories have demonstrated that calcitriol has anti-prostate cancer effects *in vitro*, including inhibition of cell proliferation, invasiveness, induction of cell cycle arrest, stimulation of apoptosis, and promotion of differentiation [e.g. 39–42]. In addition, many xenograft studies have shown that calcitriol, or metabolic precursors, or related substances reduce tumor growth and metastasis, as well as having some benefit in transgenic mouse models that spontaneously develop prostate cancer [43]. Also, a variety of epidemiological studies suggest a connection between blood levels of vitamin D and prostate cancer. For example, in patients undergoing radical prostatectomy, an association between low serum vitamin D levels and a high Gleason score has been observed [44]. At the same time, in other studies, serum levels of vitamin D and prostate cancer incidence and mortality are not strongly correlated [45]. There may be a variety of reasons for this lack of consensus, including which vitamin D metabolite was measured and the absence of information about relative VDR and 1-hydroxylase expression in the patients. To overcome the limitations of single agent calcitriol toxicity in the clinical setting, one approach has been to develop analogs of calcitriol that maintain their anti-cancer activity without producing symptoms of hypervitaminosis D [e.g. 46, 47], but an ideal candidate is still lacking. Here, our results suggest that dual therapy with S179D PRL may be a viable approach.

In addition to effects via induction of the VDR, S179D PRL has additional anti-prostate cancer benefit derived from interruption of the autocrine PRL growth loop mediated through PRLRLF signaling [5, 9]. Upon examination of a series of 80 prostate cancer specimens, autocrine prolactin immunostaining was positive in 54% of samples and local prolactin levels positively correlated with both high Gleason scores and activation of Stat5a/b, the major downstream signaling protein from the PRLRLF to growth [48]. S179D PRL inhibits Stat5 activation [11] and changes splicing of the pre-mRNA for the PRLR to increase expression of the short form 1b receptor, which in turn signals to upregulate p21, the VDR, and eventually increases apoptosis [12, 14]. Increased expression of the SF1b receptor also reduces expression of proteins associated with migration and invasion in PC3 prostate cancer cells [9]. There may also be added benefit from the stimulation of differentiation seen with S179D PRL *in vivo* in the normal rat prostate [49].

Although our use of human xenografts in nude mice allowed us to examine the impact of S179D PRL on autocrine PRL, there would have been no part of the beneficial result that was due to inhibition of effects of circulating PRL since mouse PRL does not interact with the human PRLRLF expressed on PC-3 cells [50]. One would therefore predict additional benefits of S179D PRL in a clinical situation since then it would also interfere with the prostate cancer-promoting effects of circulating PRL [51]. The full benefit of this dual therapy would therefore only be realized in a clinical setting.

In conclusion, findings from this study indicate that tumors from mice receiving dual therapy showed an increase in tumor cell death with no evidence of tissue damage akin to hypervitaminosis D. Twenty eight days of dual therapy caused 50% of the tumor cells to die. A dual therapy approach may therefore be a viable option in the treatment of CRPC.

## Figures and Tables

**Table 1:** Treatment details.

Treatment (Concentration Loaded into Alzet Pump)	Infusion Dose per hour (Rate = 0.25μl/h)	Total Dose Delivered During 28-day Treatment Period	Number of Mice at End of Study
Control: 17 ng/mL Calcitriol	4.25 pg/h Calcitriol	2.96 ng Calcitriol	n=7
S179D PRL 1mg/mL	250ng/h S179D PRL	174 μg S179D PRL	n=8
17 ng/mL Calcitriol + S179D PRL 1 mg/mL	4.25 pg/h Calcitriol + 250 ng/h S179D PRL	2.96 ng Calcitriol + 174 μg S179DPRL	n=8
170 ng/mL Calcitriol	42.5 pg/h Calcitriol	29.6 ng Calcitriol	n=8
170 ng/mL Calcitriol + S179D PRL 1 mg/mL	42.5 pg/h Calcitriol + 250 ng/h S179DPRL	29.6 ng Calcitriol + 174 μg S179DPRL	n=8
850 ng/mL Calcitriol	220 pg/h Calcitriol	148 ng Calcitriol	n=8
850 ng/mL Calcitriol + S179D PRL 1 mg/mL	220 pg/h Calcitriol + 250 ng/h S179D PRL	148 ng Calcitriol + 174 μg S179D PRL	n=8
